# Adolescent developmental task accomplishment: a descriptive analysis of Havighurst's framework among secondary-school students

**DOI:** 10.3389/fpsyg.2026.1775566

**Published:** 2026-06-10

**Authors:** Usani Joseph Ofem, Paulina Mbua Anake, Nnyenkpa Ntui Anyin, Vera Mpoun Obibessong, Cyril Bisong Abuo, Enemadukwu David Ahmed, Joseph Amushe, Princess Esther-Wanibokom Otu, James Ukatu

**Affiliations:** 1 Alex Ekwueme Federal University, Ndufu-Alike, Ndufu-Alike, Nigeria; 2 University of Calabar, Calabar, Nigeria

**Keywords:** career preparation, developmental tasks, emotional independence, family structure, gender differences, Havighurst's framework, social responsibility

## Abstract

Adolescence represents a critical stage for the acquisition of key developmental competencies essential for personal, social, and civic functioning. This study, adolescent developmental task accomplishment: a descriptive analysis of Havighurst's Framework among Secondary-School Students, investigated the extent to which Nigerian adolescents achieve Havighurst's developmental tasks across multiple domains. Employing a descriptive research design, data were collected from 2,287 secondary-school students using the validated Adolescents' Developmental Task Accomplishment Scale (ADTAS), demonstrating high reliability across subscales. Results revealed that adolescents generally accomplished most developmental tasks, including Peer Relationships, Body Acceptance, Emotional Independence, Family Life Preparation, Career Preparation, Value Orientation and Ethics, and Social Responsibility, while Gender Role Identification was least achieved. Career orientation and social responsibility were the most strongly accomplished domains, whereas peer relationships, emotional independence, family life preparation, and body acceptance were moderately attained. Analyses further indicated gendered patterns, with males excelling in autonomy, career engagement, and social participation, and females performing better in relational, ethical, and family-oriented tasks. Family structure favored adolescents from monogamous households in relational, emotional, and social tasks, while older adolescents (17–19 years) outperformed younger peers in autonomy, ethical reasoning, and family preparation. These findings underscore the relevance of Havighurst's framework in diverse cultural contexts and highlight the need for targeted interventions to support holistic adolescent development.

## 1 Introduction

Adolescence is widely recognized as a critical developmental stage characterized by rapid biological, psychological, and social transformations that shape long-term life outcomes (Steinberg, 2017). During this transitional period between childhood and adulthood, individuals confront complex developmental demands, including identity formation, emotional regulation, and the establishment of social roles. Erikson (1968) conceptualized adolescence as the stage of identity vs. role confusion, emphasizing that the successful resolution of identity-related conflicts is essential for achieving psychological stability and social integration. While this perspective provides valuable insight into the internal psychological struggles of adolescents, it does not sufficiently capture the structured expectations and environmental influences that shape developmental outcomes. Similarly, Havighurst's Developmental Task Theory identifies specific age-related tasks that adolescents are expected to accomplish, such as forming mature peer relationships, achieving emotional independence, and preparing for occupational roles (Havighurst, 1972). However, existing applications of Havighurst's framework in the literature often remain descriptive, focusing on listing developmental tasks without critically examining the mechanisms that determine whether these tasks are successfully accomplished. More critically, prior studies rarely articulate how individual and contextual variables are theoretically connected to variations in developmental task accomplishment, thereby limiting the explanatory depth of existing research.

To address this limitation, the present study adopts a multidimensional framework that integrates Havighurst's developmental task theory with Erikson's psychosocial perspective and ecological explanations of development. This integration provides a coherent basis for understanding adolescent development by linking expected developmental outcomes (Havighurst), internal psychological processes (Erikson), and environmental systems within which development occurs. In this framework, developmental task accomplishment is conceptualized not as an isolated outcome but as the result of interactions between individual characteristics and contextual conditions. Ecological perspectives emphasize that adolescents are embedded within interconnected systems such as family, school, peer groups, and broader cultural contexts, all of which shape developmental opportunities and constraints (Santrock, 2019). Within this integrated framework, demographic variables such as gender, age, and family type are not treated as mere background characteristics but as theoretically meaningful factors that are associated with differences in developmental experiences and outcomes. This provides a clear conceptual linkage between the independent variables and developmental task accomplishment as the outcome of interest.

The Nigerian context presents a particularly compelling setting for examining adolescent development due to the complex interplay of socio-cultural, economic, and institutional factors that shape youth experiences. Adolescents in Nigeria are expected to meet universal developmental tasks, yet their ability to do so is often constrained by structural inequalities, cultural expectations, and systemic challenges. For instance, cultural norms regulating gender interactions may limit opportunities for developing mature peer relationships, while deficiencies in the educational system hinder vocational preparation and career development (Odo, 2015; Okafor and Amayo, 2020). Additionally, family instability, economic hardship, and limited access to support systems can affect emotional independence and identity formation (Adegoke, 2015). These realities suggest that developmental task accomplishment is not solely determined by individual effort but is associated with contextual conditions. In this study, family type is operationally defined as the form of family arrangement in which the adolescent resides or was raised, categorized into nuclear, single-parent, and extended family systems. Drawing from ecological theory, family type represents a core microsystem that shapes access to emotional support, supervision, and role modeling, which are essential for successful developmental task accomplishment.

Conceptually, gender, age, and family type are linked to developmental task accomplishment through established theoretical pathways. Gender is associated with developmental outcomes through differential socialization processes that shape behavioral expectations, role performance, and opportunities for task mastery within specific cultural contexts (Hyde, 2014). Age is inherently connected to developmental task accomplishment because developmental tasks are age-graded; progression through adolescence is associated with increasing cognitive maturity, emotional regulation, and social competence, which are necessary for successful task completion (Steinberg, 2017). Family type, as part of the adolescent's immediate environment, influences access to emotional support, supervision, and role modeling, all of which shape developmental outcomes (Amato, 2005). These theoretical connections establish that variations in developmental task accomplishment can be meaningfully examined through differences in gender, age, and family type, thereby strengthening the conceptual alignment of the study variables.

Despite growing scholarly attention to adolescent development, existing studies in Nigeria remain limited in several important ways. Many studies focus on isolated dimensions of development, such as academic performance, peer relations, or moral behavior, without recognizing the interconnected nature of developmental tasks. While such studies provide valuable insights, they do not offer a comprehensive understanding of how multiple developmental domains interact. More importantly, there is a clearly identifiable empirical gap in the literature: the absence of multivariate, theory-driven studies that examine how sociodemographic variables such as gender, age, and family type are simultaneously associated with variations in overall developmental task accomplishment. Most existing studies rely on univariate and descriptive approaches, which do not adequately capture combined patterns of variation across developmental domains. This limitation has resulted in fragmented knowledge and weak theoretical integration. Furthermore, many studies fail to explicitly connect demographic variables to developmental outcomes within a coherent theoretical framework. This study therefore addresses a sharply defined problem: the lack of integrated, theoretically grounded, and multivariate evidence explaining differences in developmental task accomplishment among adolescents. Accordingly, the study aims to examine the extent of developmental task accomplishment and differences in developmental task accomplishment based on gender, age, and family type among adolescents, thereby providing a more comprehensive and theoretically grounded understanding of adolescent development within the Nigerian context.

### 1.1 Study questions

1. What patterns of developmental task accomplishment exist among adolescents across developmental domains?
2. What differences exist in adolescents' developmental task accomplishment based on gender, family type, and age?
3. What is the interactuve effect of age and gender in adolescents' developmental task accomplishment?

## 2 Review of literature

### 2.1 Empirical review of adolescent developmental tasks

Adolescent development has been widely conceptualized within Robert Havighurst's developmental task framework, which posits that individuals must accomplish specific tasks at different life stages to ensure successful transition into adulthood. Among adolescents, these tasks include developing mature peer relationships, achieving gender role identity, accepting one's physical body, attaining emotional independence, preparing for family life and economic careers, establishing a value system, and assuming social responsibility. While these domains are theoretically presented as distinct, contemporary scholarship increasingly emphasizes their interconnected nature, suggesting that adolescent development is multidimensional and shaped by complex interactions between individual, social, and cultural factors.

Empirical research on peer relationship development consistently highlights its centrality in adolescent psychosocial adjustment. Studies in Western contexts demonstrate that supportive peer networks are associated with improved academic engagement, emotional wellbeing, and reduced risk behaviors (Brown and Larson, 2009; Wentzel et al., 2016). Similarly, cross-cultural research indicates that peer relationships function as a critical context for identity exploration and social learning. However, the nature of these relationships varies across cultures. In collectivist societies, conformity and group harmony tend to shape peer interactions, whereas individualistic contexts emphasize autonomy and self-expression (Fuligni and Hardway, 2004). Evidence from Nigeria suggests that adolescents often navigate tensions between peer expectations and parental authority, particularly in contexts where cultural norms regulate social interactions (Okpala and Umeh, 2020). Despite these insights, existing studies remain largely descriptive and fail to examine how peer relationships interact with other developmental domains or how contextual pressures such as digital exposure and socioeconomic constraints shape peer dynamics.

Gender role identification represents another critical but complex developmental domain. Research across cultural contexts demonstrates that adolescence is a sensitive period for consolidating gender identity due to biological changes, cognitive development, and increased social expectations (Steensma et al., 2013). In many African societies, rigid gender norms continue to influence adolescents' roles and aspirations, often reinforcing traditional expectations related to masculinity and femininity (Nwoye, 2015). Western studies similarly highlight the persistence of gender stereotypes, particularly in shaping academic motivation and career aspirations (Eccles, 1999). At the same time, emerging evidence suggests increasing fluidity in gender identity, especially in contexts exposed to globalization and media influences (Galambos et al., 2009). However, much of the literature treats gender role identification in isolation, with limited attention to how it interacts with other developmental tasks such as career preparation, emotional independence, and value formation. In Nigeria, empirical studies remain scarce, leaving significant gaps in understanding how adolescents negotiate gender identity within competing traditional and modern influences.

Body acceptance is closely linked to adolescents' self-concept and psychological wellbeing. Research indicates that positive body image is associated with higher self-esteem, better peer relationships, and lower risk of mental health challenges (Harter, 2012; Tiggemann and Slater, 2014). Conversely, negative body perception has been linked to depression, anxiety, and unhealthy behaviors such as disordered eating (Meland et al., 2007). The increasing influence of social media has intensified body image concerns globally, as adolescents are exposed to idealized and often unrealistic standards of beauty (Perloff, 2014). While participation in physical activities has been shown to enhance body confidence and social acceptance (Fredricks and Eccles, 2006), negative peer experiences such as teasing can undermine these benefits (Slater and Tiggemann, 2011). Despite extensive research in Western contexts, limited evidence exists on how African adolescents experience body image within the intersection of traditional values and global media influences. In Nigeria, where cultural perceptions of body image are rapidly evolving, there is a need for empirical studies that examine how body acceptance interacts with identity formation and social adjustment.

Emotional independence is another key developmental task that reflects adolescents' growing autonomy and self-regulation. Self-determination theory emphasizes that autonomy is essential for psychological growth, as individuals internalize values and make decisions based on personal convictions (Ryan and Deci, 2006). Parenting styles play a critical role in this process, with supportive environments fostering independence and authoritarian approaches often constraining it (Soenens and Vansteenkiste, 2010). Adolescents who develop emotional independence are more likely to demonstrate resilience, effective decision-making, and adaptive coping strategies (Zimmer-Gembeck and Skinner, 2011). However, contemporary research highlights new challenges associated with digital dependency, where adolescents may appear independent offline but remain emotionally reliant on online validation. This emerging dimension suggests that traditional conceptualizations of autonomy may require re-evaluation in the context of modern technological environments. Notably, much of the existing literature is rooted in Western contexts, leaving limited understanding of how emotional independence is shaped within African cultural systems that emphasize interdependence and family cohesion.

Preparation for family life reflects adolescents' readiness to assume future roles related to marriage, parenting, and domestic responsibilities. Studies show that adolescents' attitudes toward family life are shaped by cultural norms, parental modeling, and social expectations (Raley and Sweeney, 2020). In collectivist societies, family formation is often viewed as a central life goal, whereas individualistic cultures may prioritize personal development before family commitments (Arnett, 2014). In sub-Saharan Africa, early marriage remains a significant concern, particularly for girls, with implications for education and long-term wellbeing (UNICEF, 2021). Research indicates that adolescents who lack exposure to structured family life education may enter adulthood unprepared for relational and parental responsibilities (Holman and Oswald, 2016). Despite its importance, this domain remains underexplored among adolescents, particularly in Nigerian contexts where most studies focus on adult populations rather than early developmental preparation.

Career preparation has received considerable attention in developmental research, with evidence highlighting the importance of early vocational exploration in shaping future employment outcomes (Lent et al., 1994). Adolescents' career aspirations are influenced by factors such as parental expectations, socioeconomic background, and access to educational opportunities (Sawitri et al., 2014). School-based interventions, including career counseling and experiential learning, have been shown to enhance vocational readiness and decision-making (Galliott and Graham, 2015). However, disparities in access to resources remain a critical issue, particularly in developing contexts where limited infrastructure and high unemployment constrain opportunities (Organisation for Economic Co-operation and Development (OECD), 2018). Gender stereotypes also continue to influence career choices, reinforcing occupational segregation (Patton and McMahon, 2014). In Nigeria, research on career preparation is limited and often fails to examine how adolescents navigate career development within the broader context of developmental tasks, highlighting a need for integrated approaches.

Value orientation and ethical development represent foundational aspects of adolescent growth, shaping decision-making and social behavior. Kohlberg's (1981) theory of moral development outlines a progression from externally driven reasoning to more autonomous ethical judgment. Empirical studies indicate that moral development is influenced by education, peer interaction, and cultural context (Nucci, 2016). While collectivist cultures often emphasize conformity to social norms, individualistic societies encourage independent moral reasoning (Hardy and Carlo, 2011). However, critiques of moral development theory argue that much of the evidence is based on Western populations, raising questions about cultural universality (Miller et al., 2014). In Nigeria, limited research exists on how adolescents integrate traditional ethical systems with contemporary influences such as globalization and digital media, suggesting a significant gap in understanding moral development within this context.

Social responsibility, as a developmental task, reflects adolescents' growing engagement with their communities and broader society. Research shows that participation in civic activities enhances self-esteem, social competence, and long-term engagement in community life (Zaff et al., 2010). However, expressions of social responsibility vary across cultural contexts. In collectivist societies, civic engagement is often tied to family and community obligations, while in individualistic settings it may take the form of voluntary service or activism (Lawford et al., 2013). In sub-Saharan Africa, structural challenges such as poverty and limited institutional support shape how adolescents engage in civic activities. Despite its importance, research in Nigeria remains limited, particularly in examining how social responsibility interacts with other developmental domains.

Overall, the reviewed literature reveals important gaps that justify the present study. First, existing research is largely fragmented, focusing on individual developmental tasks rather than examining their interconnected nature. Second, there is a lack of holistic analyses that consider how demographic variables such as gender, age, and family structure jointly influence developmental outcomes. Third, limited attention has been given to rural and context-specific experiences, particularly within Nigerian settings. Finally, there is a lack of integrated theoretical frameworks that combine developmental task theory with broader psychosocial and ecological perspectives. Beyond these identified gaps, the literature provides a clear theoretical and empirical basis for examining differences in developmental task accomplishment across key sociodemographic variables. Drawing from Havighurst Developmental Task Theory, developmental tasks are age-graded and require progressive levels of cognitive, emotional, and social competence, suggesting that adolescents at different ages may demonstrate varying levels of task accomplishment. Similarly, insights from Erikson's Psychosocial Development Theory indicate that identity formation and role development are influenced by social expectations and role experiences, which are often differentiated along gender lines (Hyde, 2014; Eccles, 1999). From an ecological perspective, Bronfenbrenner's Ecological Systems Theory highlights the central role of the family as a microsystem shaping adolescents' access to emotional support, supervision, and role modeling, which are critical for developmental outcomes (Amato, 2005). These theoretical and empirical insights collectively suggest that developmental task accomplishment may not be uniform across adolescents but may vary based on gender, age, and family type.

### 2.2 Current study and hypotheses development

The body of literature reviewed indicates that adolescents' developmental task accomplishment is a complex and multidimensional phenomenon shaped by interacting psychological, social, and environmental influences. Although extant studies have investigated individual developmental domains such as peer interaction, emotional autonomy, vocational preparation, and moral orientation, much of the existing scholarship remains compartmentalized and predominantly descriptive in orientation. Most prior investigations examine these developmental dimensions independently, with limited attention given to their interconnected nature or to the combined influence of sociodemographic characteristics on developmental outcomes. In addition, existing studies frequently adopt narrow analytical approaches that overlook the broader psychosocial and ecological conditions within which adolescent development occurs. As a result, there remains insufficient multivariate and theoretically integrated evidence explaining how demographic variables contribute to differences in developmental task accomplishment across multiple developmental domains.

**Figure 1:**
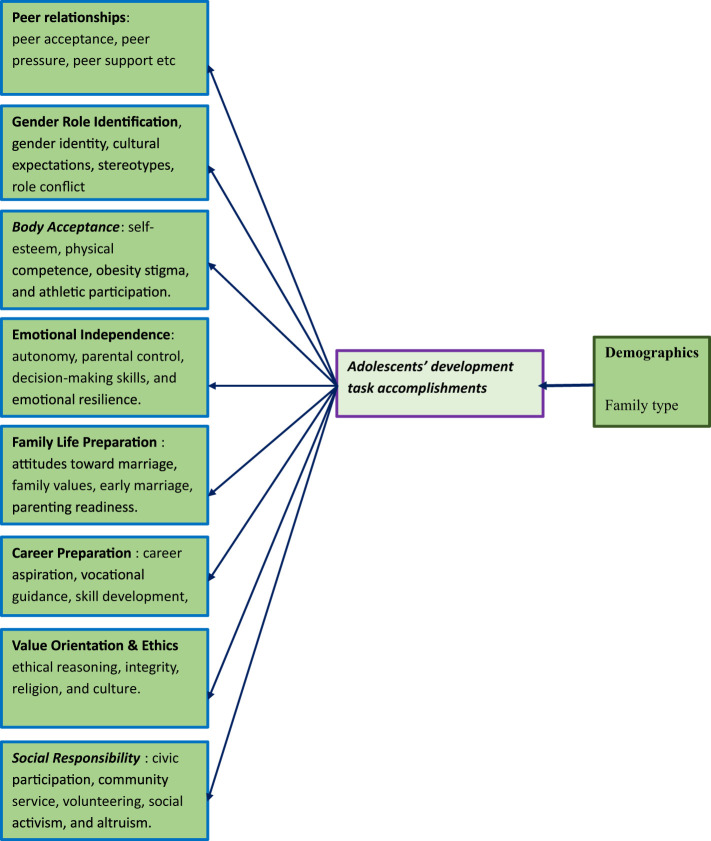
Conceptual model of adolescent's developmental task.

To address these limitations, the present study is anchored in an integrated conceptual framework (see Figure 1) derived from Havighurst's Developmental Task Theory, Erikson's Psychosocial Theory, and Bronfenbrenner's Ecological Systems Theory. Within this framework, adolescents' developmental task accomplishment constitutes the primary outcome variable and is conceptualized as being shaped by several interrelated developmental domains, including peer relationships, gender role identification, body acceptance, emotional independence, family life preparation, career preparation, value orientation and ethics, and social responsibility. The framework further conceptualizes gender, age, and family type as theoretically relevant demographic variables capable of influencing developmental outcomes through their impact on adolescents' experiences, opportunities, and social expectations.

From Erikson's psychosocial standpoint and broader socialization perspectives, developmental differences based on gender are anticipated because male and female adolescents are often exposed to different cultural expectations, behavioral norms, and role experiences that shape their developmental trajectories (Hyde, 2014). Likewise, Havighurst's framework posits that developmental tasks are inherently age-related, implying that progression through adolescence is associated with increasing levels of emotional regulation, social competence, and psychological maturity necessary for effective task accomplishment (Steinberg, 2017). Furthermore, Bronfenbrenner's ecological theory identifies the family as a critical microsystem influencing adolescents' developmental experiences through the provision of emotional support, supervision, and role modeling. Consequently, adolescents raised within different family structures may encounter varying developmental conditions and opportunities that affect developmental adjustment (Amato, 2005).

Beyond the direct influence of these demographic variables, developmental theories further suggest that adolescent outcomes may emerge from the interaction between sociodemographic characteristics. In particular, the effect of gender on developmental task accomplishment may not remain constant across age groups because developmental expectations, psychosocial challenges, and socialization experiences evolve as adolescents mature. Therefore, differences associated with gender may become more pronounced or diminish depending on adolescents' developmental stage. Against this background, the present study investigates both the independent and interactive effects of gender, age, and family type on adolescents' developmental task accomplishment. Guided by the conceptual framework and empirical evidence reviewed, the study formulated the following hypotheses.

### 2.3 Hypotheses development

**H1:** Adolescents' developmental task accomplishment differs significantly based on gender.
**H2:** Adolescents' developmental task accomplishment differs significantly across age groups.
**H3:** Adolescents' developmental task accomplishment differs significantly based on family type.
**H4:** There is a significant interaction effect of gender and age on adolescents' developmental task accomplishment.

## 3 Materials and methods

### 3.1 Study procedure and participants

The study employed a descriptive survey design, deemed suitable for assessing adolescents' developmental task accomplishment within a natural school context without manipulating variables (Creswell, 2014). This design is particularly appropriate because the study seeks to describe existing conditions, examine relationships among variables, and compare group differences (e.g., gender, age, and family structure) without any form of experimental intervention. It allows for the collection of data from a large population at a single point in time, thereby providing a realistic representation of adolescents' developmental status as it naturally occurs. Furthermore, the descriptive survey design aligns with the study's quantitative approach, enabling the use of statistical techniques to analyze patterns and variations in developmental task accomplishment.

The target population consisted of 21,763 in-school adolescents aged 11–19 years in the Calabar Education Zone, providing diversity across age, gender, and family background (Fraenkel and Wallen, 2009). The sampling frame comprised officially registered secondary schools within the Calabar Education Zone as obtained from the State Ministry of Education, ensuring that all eligible participants had a known and non-zero chance of selection. Inclusion criteria required that participants be enrolled secondary school students within the specified age range, while those outside the age bracket or not formally enrolled were excluded to maintain consistency and relevance to the study objectives.

A multistage sampling approach was adopted. The seven educational authorities served as strata, from which 76 schools were identified. Fifteen schools (20%) were proportionately selected, with a combined enrollment of 7,622 students. This stage involved proportionate stratified random sampling to ensure that schools from each educational authority were adequately represented. From this, 30% (2,287 students) formed the sample, chosen through systematic random sampling to ensure equal representation (Cohen et al., 2018). The choice of 30% of the accessible population is considered adequate for survey studies of this nature, as it enhances statistical power, improves representativeness, and reduces sampling error. The systematic random sampling technique ensured that every nth student had an equal probability of selection, thereby minimizing selection bias.

Respondents comprised 52% males (1,189) and 48% females (1,098). Age distribution indicated 25% were 10–13 years, 45% were 14–16 years, and 30% were 17–19 years. Family background showed 40% from polygamous and 60% from monogamous households. These distributions suggest that the sample is reasonably representative of the population in terms of key demographic variables, thereby enhancing the generalizability of the findings. The use of multistage and probability-based sampling techniques further strengthens the external validity of the study. Data collection commenced after obtaining ethical approval from the Institutional Review Board (IRB) of Alex Ekwueme Federal University (Approval No. FUNAI/2024/1882). Formal letters of introduction from the Board granted access to participating schools, and permission was secured from each school's administration. The study's purpose was explained to students, who were informed that participation was voluntary. To ensure consistency, five trained research assistants administered the questionnaire. A total of 2,287 students received the instrument over a 2-week period, allowing sufficient time for completion. Of these, 2,102 questionnaires were returned, yielding a 91.9% response rate. After screening for completeness, 1,982 valid responses were retained for analysis.

### 3.2 Measures

The instrument used for data collection was a structured questionnaire titled *Adolescents' Developmental Task Accomplishment Scale (ADTAS)*, developed by the researchers after a careful review of relevant literature and consultation with experts in adolescent psychology and education to ensure content validity. The questionnaire consisted of two sections. Section A sought demographic information such as gender, age, family type, and school type, measured with categorical options. It also included an item where participants indicated their willingness to take part in the study.

Section B was designed to measure Havighurst's (1972) eight developmental tasks, with each construct represented by five items and responses recorded on a four-point frequency scale ranging from Never (1) to Often (4). Peer relationships were operationally defined as adolescents' ability to initiate, maintain, and sustain supportive and meaningful interactions with peers, including friendship quality, acceptance, cooperation, and resistance to negative peer pressure within social contexts that promote mutual trust and shared activities. Sample item: “Spend time helping a friend solve a personal problem.” Gender role identification was operationally defined as adolescents' understanding, acceptance, and expression of socially and culturally defined roles associated with masculinity and femininity, including identity formation, role expectations, and stereotype negotiation. Sample item: “Take on a role in a school activity that aligns with your interests, regardless of gender.” Body acceptance was operationally defined as the extent to which adolescents develop a positive perception of their physical appearance, body image, and physical competence, including self-esteem and resilience against societal pressures. Sample item: “Accept your body's strengths and limitations in class activities.” Emotional independence was operationally defined as adolescents' ability to regulate emotions, make autonomous decisions, and demonstrate self-reliance without excessive dependence on parental or external approval. Sample item: “Make a decision about a personal project without asking parents.” Family life preparation was operationally defined as adolescents' readiness to assume future family roles, including attitudes toward marriage, parenting, and responsibilities within the family unit. Sample item: “Plan activities that involve helping family members.” Career preparation was operationally defined as the extent to which adolescents develop awareness, aspirations, and competencies related to future occupational roles through engagement in career exploration and skill development activities. Sample item: “Participate in a career guidance session.” Value orientation and ethics were operationally defined as the extent to which adolescents internalize moral principles, cultural values, and ethical standards that guide behavior and decision-making. Sample item: “Participate in a class debate on ethical issues.” Finally, social responsibility was operationally defined as adolescents' active involvement in behaviors that promote the welfare of others and the community, including civic participation, volunteering, and altruistic actions. Sample item: “Participate in campaigns for social causes at school.”

### 3.3 Content validity

The validity of the instrument was established through expert review in educational psychology, measurement and evaluation, and adolescent development. The experts assessed the instrument against the study objectives and rated each item on a four-point scale ranging from “relevant” to “highly relevant.” Based on these ratings, the Item Content Validity Index (I-CVI) and Scale Content Validity Index (S-CVI) were computed. The I-CVI values across the evaluation criteria—precision, clarity, and suitability—ranged from 0.78 to 0.89, while the S-CVI values ranged from 0.89 to 0.96, meeting recommended standards for content validity (Polit and Beck, 2006; Lynn, 1986). These results indicate a high level of expert agreement regarding the relevance, clarity, and representativeness of the instrument items, thereby providing strong evidence of content validity. To further substantiate this, the I-CVI values across the three evaluation criteria—precision, clarity, and suitability—alongside the corresponding S-CVI and reliability coefficients are presented in Table 1.

**Table 1:** Content validity and reliability indices of the instrument.

S/N	Instrument Subscale	Precision (I-CVI)	Clarity (I-CVI)	Suitability (I-CVI)	S-CVI	(α)
1	Peer relationships	0.84	0.86	0.88	0.90	0.82
2	Gender role identification	0.81	0.83	0.85	0.89	0.79
3	Body acceptance	0.80	0.82	0.84	0.89	0.78
4	Emotional independence	0.85	0.87	0.89	0.92	0.86
5	Family life preparation	0.78	0.80	0.82	0.89	0.76
6	Career preparation	0.83	0.85	0.87	0.91	0.84
7	Value orientation and ethics	0.86	0.88	0.89	0.94	0.89
8	Social responsibility	0.84	0.86	0.88	0.93	0.87

### 3.4 Construct validity

A pilot study conducted with 150 in-school adolescents outside the main study area. Descriptive statistics of the measured constructs indicated that the mean scores as presented in Table 2 ranged from 2.88 to 3.12, with corresponding standard deviations ranging from 0.58 to 0.74, suggesting moderate variability in responses across the factors. The distribution of the data further showed that skewness values ranged from −0.61 to −0.18, while kurtosis values ranged from −0.72 to 0.64, indicating that the data approximated normal distribution and met the assumptions for factor analysis.

**Table 2:** Exploratory factor analysis (EFA) results of the instrument.

S/N	Factor	Mean	SD	Skewness	Kurtosis	λ	*λ^2^*	ε
1	Peer relationships	2.94–3.08	0.61–0.72	−0.58 to −0.22	−0.68 to 0.61	0.75–0.89	0.56–0.79	0.21–0.44
2	Gender role identification	2.88–3.01	0.64–0.73	−0.61 to −0.25	−0.72 to 0.58	0.74–0.87	0.55–0.76	0.24–0.45
3	Body acceptance	2.90–3.05	0.60–0.71	−0.55 to −0.21	−0.69 to 0.60	0.76–0.88	0.58–0.77	0.23–0.42
4	Emotional independence	2.97–3.12	0.58–0.70	−0.49 to −0.18	−0.64 to 0.64	0.78–0.91	0.61–0.83	0.17–0.39
5	Family life preparation	2.89–3.02	0.63–0.74	−0.57 to −0.23	−0.71 to 0.59	0.74–0.86	0.55–0.74	0.26–0.45
6	Career preparation	2.95–3.10	0.59–0.71	−0.52 to −0.20	−0.66 to 0.62	0.77–0.90	0.59–0.81	0.19–0.41
7	Value orientation and ethics	2.96–3.11	0.58–0.69	−0.50 to −0.19	−0.65 to 0.63	0.79–0.91	0.62–0.83	0.17–0.38
8	Social responsibility	2.59–2.98	0.51–072	−0.48 to −0.20	0.61–0.50	0.78–0.87	0.61 to 0.76	0.22–0.13

Following this, the suitability of the dataset for Exploratory Factor Analysis (EFA) was confirmed, as the Kaiser–Meyer–Olkin (KMO) measure of sampling adequacy was 0.91, indicating excellent adequacy, while Bartlett's Test of Sphericity was statistically significant (χ^2^ = 4126.37, df = 378, *p* < 0.001), confirming that the correlation matrix was appropriate for factor extraction. Using Principal Axis Factoring with Promax rotation, a seven-factor structure consistent with the theoretical subscales was extracted. The results showed that all items loaded appropriately on their respective factors, with factor loadings ranging from 0.74 to 0.91, thereby exceeding the recommended threshold and confirming strong item-factor relationships without problematic cross-loadings. The analysis further revealed that the total variance explained by the seven factors was 71.84%, with individual factor contributions ranging from 7.86% to 12.94%, indicating that the extracted factors collectively accounted for a substantial proportion of the variance in the dataset.

#### 3.4.1 Measurement model assessment

The reliability and validity of the instrument were assessed using Cronbach's alpha, composite reliability (CR), average variance extracted (AVE), and discriminant validity measures. The result as presented in Table 3 showed that Cronbach's alpha values ranged from 0.76 to 0.89, while composite reliability values ranged from 0.83 to 0.91, confirming satisfactory internal consistency. The AVE values ranged from 0.57 to 0.72, exceeding the recommended threshold of 0.50, thereby establishing convergent validity. Discriminant validity was assessed using the Fornell–Larcker criterion and HTMT ratio. The square roots of AVE (bold diagonal elements) were greater than the inter-construct correlations, while HTMT values (upper diagonal) ranged from 0.55 to 0.83, remaining below the threshold of 0.85. These results confirm that the instrument demonstrates adequate reliability, convergent validity, and discriminant validity.

**Table 3:** Reliability, convergent validity, and discriminant validity.

Construct	α	CR	AVE	PR	GRI	BA	EI	FLP	CP	VOE	SR
PR	0.82	0.87	0.62	**0.79**	0.61	0.58	0.66	0.57	0.69	0.73	0.71
GRI	0.79	0.85	0.59	0.52	**0.77**	0.55	0.63	0.60	0.65	0.70	0.67
BA	0.78	0.84	0.58	0.49	0.46	**0.76**	0.59	0.54	0.61	0.64	0.62
EI	0.86	0.90	0.69	0.55	0.51	0.48	**0.83**	0.65	0.72	0.75	0.74
FLP	0.76	0.83	0.57	0.47	0.50	0.45	0.53	**0.75**	0.68	0.71	0.69
CP	0.84	0.89	0.66	0.58	0.54	0.50	0.60	0.56	**0.81**	0.78	0.76
VOE	0.89	0.91	0.72	0.61	0.57	0.52	0.63	0.59	0.65	**0.85**	0.83
SR	0.87	0.90	0.70	0.59	0.55	0.51	0.61	0.57	0.64	0.68	**0.84**

Diagonal elements (bold) represent the square root of average variance extracted (√AVE); values below the diagonal represent inter-construct correlations, while values above the diagonal represent Heterotrait–Monotrait Ratio (HTMT). PR, peer relationships; GRI, gender role identification; BA, body acceptance; EI, emotional independence; FLP, family life preparation; CP, career preparation; VOE, value orientation and ethics; SR, social responsibility.

#### 3.4.2 Confirmatory factor analysis (CFA)

Confirmatory factor analysis (CFA) was conducted using a covariance-based structural equation modeling approach to validate the factor structure previously established through exploratory factor analysis (EFA). The analysis assessed the extent to which the observed data fit the hypothesized measurement model comprising eight latent constructs: peer relationships, gender role identification, body acceptance, emotional independence, family life preparation, career preparation, value orientation and ethics, and social responsibility.

The results indicated that the measurement model demonstrated a good and acceptable fit to the data. Specifically, the chi-square to degrees of freedom ratio (χ^2^/df) was 2.41, which is below the recommended threshold of 3.00. The incremental fit indices were satisfactory, with the comparative fit index (CFI) = 0.93, Tucker–Lewis index (TLI) = 0.92, and normed fit index (NFI) = 0.92, all exceeding the minimum criterion of 0.90. Similarly, the absolute fit indices showed that the root mean square error of approximation (RMSEA) = 0.052 and the standardized root mean square residual (SRMR) = 0.047 were within acceptable limits. Additionally, the goodness-of-fit index (GFI) = 0.91 and adjusted goodness-of-fit index (AGFI) = 0.90 further confirmed model adequacy. All standardized factor loadings as presented in Table 4 were significant and ranged from 0.74 to 0.90, confirming strong measurement properties and supporting the validity and reliability of the instrument.

**Table 4:** Model fit indices for confirmatory factor analysis.

Fit index	Recommended threshold	Obtained value	Decision
χ^2^/df	< 3.00	2.41	Acceptable fit
CFI	≥0.90	0.93	Good fit
TLI	≥0.90	0.92	Good fit
RMSEA	≤ 0.08	0.052	Good fit
SRMR	≤ 0.08	0.047	Good fit
GFI	≥0.90	0.91	Good fit
AGFI	≥0.90	0.90	Acceptable fit
NFI	≥0.90	0.92	Good fit

### 3.5 Data analysis

The data generated from the study were coded and analyzed using the Statistical Package for the Social Sciences (SPSS) version 28.0. Prior to conducting the main statistical analyses, the dataset underwent preliminary screening procedures to identify missing data, possible entry errors, outliers, and violations of assumptions associated with parametric statistical techniques. These preliminary checks were necessary to ensure the accuracy, consistency, and suitability of the data for subsequent analyses.

To ascertain the psychometric quality of the instrument, both exploratory factor analysis (EFA) and confirmatory factor analysis (CFA) were conducted. EFA was initially employed to explore the underlying factor structure of the developmental task accomplishment scale and determine the dimensional adequacy of the instrument. The appropriateness of the data for factor analysis was verified using the Kaiser–Meyer–Olkin (KMO) measure of sampling adequacy together with Bartlett's Test of Sphericity. Following this procedure, CFA was performed to validate the proposed measurement model and confirm whether the observed data adequately fit the hypothesized factor structure. In addition, convergent validity was assessed through average variance extracted (AVE) and composite reliability (CR), whereas discriminant validity was evaluated using the Fornell–Larcker criterion and the Heterotrait–Monotrait Ratio (HTMT).

Furthermore, assumption testing was carried out before the inferential analyses to confirm the appropriateness of applying parametric statistical procedures such as independent samples *t*-test and analysis of variance (ANOVA). Normality was assessed using skewness and kurtosis statistics in conjunction with visual inspection of the distribution plots. The findings revealed that all variables approximated normal distribution, with skewness and kurtosis values falling within the acceptable threshold of ±1.0 (George and Mallery, 2010). The large sample size (*N* = 1,982) further enhanced the robustness of the analyses in line with the Central Limit Theorem (Field, 2018). Homogeneity of variance was examined using Levene's Test, and the results indicated that the assumption was substantially satisfied. Descriptive statistics were used to answer the research questions, while independent samples *t*-test, one-way ANOVA, and two-way ANOVA were employed to test the hypotheses at the.05 level of significance. Effect sizes were also reported using Cohen's *d* and partial eta squared (η^2^).

## 4 Result

### 4.1 Patterns accomplishment of Havighurst's developmental tasks

To determine the overall level of accomplishment of Havighurst's developmental tasks among adolescents, the study examined eight domains: peer relationships, gender role identification, body acceptance, emotional independence, family life preparation, career preparation, value orientation and ethics, and social responsibility. Mean scores and standard deviations were used for analysis, with results summarized in Table 5.

**Table 5:** Mean rating and standard deviation level of accomplishment of Havighurst's developmental tasks among secondary-school adolescents in Nigeria.

Construct	Task-oriented item	Mean	SD	Remarks
Peer relationships	1. Spend time helping a friend solve a personal problem.	3.01	1.01	Achieved
	2. Join a group activity with peers.	3.22	0.90	Achieved
	3. Encourage a friend who is feeling left out.	2.48	0.45	Not achieved
	4. Stand up to peers if they pressure you to do something wrong.	3.11	0.14	Achieved
	5. Participate in a team project with classmates.	2.89	0.65	Achieved
	Aggregate score	2.94	0.63	Achieved
Gender role identification	1. Take on a role in a school activity that aligns with your interests, regardless of gender.	2.30	0.92	Not achieved
	2. Discuss with peers or teachers what you feel about gender expectations.	2.11	0.78	Not achieved
	3. Challenge a stereotype you encounter at school.	2.01	0.80	Not achieved
	4. Choose an activity based on your preference, not societal expectations.	3.11	0.45	Achieved
	5. Express your personal style or hobbies confidently.	2.71	0.50	Achieved
	Aggregated score	2.44	0.69	Not achieved
Body acceptance	1. Participate in a sport or physical activity you enjoy.	3.10	0.16	Achieved
	2. Compliment a friend on their physical abilities.	2.19	1.01	Not achieved
	3. Set a personal goal for improving a physical skill.	3.00	0.28	Achieved
	4. Attend a school fitness or wellness event.	2.76	0.35	Achieved
	5. Accept your body's strengths and limitations in class activities.	2.68	0.40	Achieved
	Aggregated score	2.75	0.44	Achieved

Decision rule: a criterion mean of 2.50, derived from the midpoint of the 4-point Likert scale, was used as the threshold for Interpretation. Mean scores below 2.50 were classified as “not achieved,” scores from 2.50 to 2.99 as “moderately achieved,” and scores of 3.00 and above as “highly achieved.”

The findings on peer relationships revealed that adolescents performed above the criterion mean of 2.50, with an overall mean of 2.94 (SD = 0.63). This indicates that peer-related tasks were generally achieved. The highest mean item, “*Join a group activity with peers*” (*M* = 3.22, SD = 0.90), shows that adolescents actively engage in group activities, reflecting a strong inclination toward social participation. The next strongest indicator, “*Stand up to peers if they pressure you to do something wrong*” (*M* = 3.11, SD = 0.14), suggests reasonable assertiveness in resisting negative peer influence. However, the lowest-scoring item, “*Encourage a friend who is feeling left out*” (*M* = 2.48, SD = 0.45), fell slightly below the criterion mean. This indicates a gap in providing emotional support, suggesting adolescents may be less confident in empathizing with socially excluded peers.

In terms of gender role identification, the aggregate mean was 2.44 (SD = 0.69), which is marginally below the criterion mean, suggesting moderate but incomplete accomplishment of this task. The highest item, “*Choose an activity based on your preference, not societal expectations*” (*M* = 3.11, SD = 0.45), shows that adolescents are somewhat confident in making independent choices outside gender norms. The second-highest, “*Express your personal style or hobbies confidently*” (*M* = 2.71, SD = 0.50), indicates moderate comfort in self-expression. However, the lowest-scoring item, “*Challenge a stereotype you encounter at school*” (*M* = 2.01, SD = 0.80), highlights that most adolescents find it difficult to confront stereotypes, pointing to a need for interventions that encourage critical reflection and confidence in addressing cultural expectations.

For body acceptance, adolescents scored above the criterion mean, with an aggregate mean of 2.75 (SD = 0.44). The strongest item was “*Participate in a sport or physical activity you enjoy*” (*M* = 3.10, SD = 0.16), indicating confidence and satisfaction in physical engagement. The next strongest, “*Set a personal goal for improving a physical skill*” (*M* = 3.00, SD = 0.28), reflects motivation and personal development. Conversely, the lowest item, “*Compliment a friend on their physical abilities*” (*M* = 2.19, SD = 1.01), fell short of the benchmark, suggesting limited peer encouragement in physical competence.

Overall, adolescents demonstrated stronger achievement in tasks involving group participation, making independent activity choices, and engaging in enjoyable physical activities. However, weaker areas emerged in providing empathy to peers, challenging gender stereotypes, and recognizing others' abilities. These results suggest that while adolescents are progressing well in self-directed and participatory domains, targeted support in empathy, stereotype resistance, and peer recognition could enhance developmental outcomes further.

As presented in Table 6, the overall emotional independence of adolescents was achieved, with an aggregate mean of 2.91 (SD = 0.53), which is above the criterion mean of 2.50. The strongest indicator was “*Express your opinion in class discussions*” (*M* = 3.15, SD = 0.60), showing that students are confident in voicing their ideas in academic settings. The second-highest item, “*Solve a problem independently at school*” (*M* = 3.04, SD = 0.55), reflects emerging autonomy and problem-solving skills. Since all items exceeded the benchmark, the findings suggest that adolescents are generally self-reliant, able to make decisions, and adaptable to new situations. This highlights growth in autonomy and resilience, although unmeasured areas such as conflict management may still require targeted support.

**Table 6:** Mean rating and standard deviation level of accomplishment of Havighurst's developmental tasks among secondary-school adolescents in Nigeria.

Construct	Task-oriented item: how often do you do the following?	Mean	SD	Remarks
Emotional independence	1. Make a decision about a personal project without asking parents.	2.78	0.50	Achieved
	2. Solve a problem independently at school.	3.04	0.55	Achieved
	3. Express your opinion in class discussions.	3.15	0.60	Achieved
	4. Manage your time to complete a task without reminders.	2.55	0.48	Achieved
	5. Adapt to unexpected changes in a group project.	3.01	0.52	Achieved
	Aggregated Score	2.906	0.53	Achieved
Family life preparation	1. Discuss family values or expectations with peers.	3.21	0.55	Achieved
	2. Plan activities that involve helping family members.	3.01	0.50	Achieved
	3. Role-play scenarios of parenting or caring for younger siblings.	2.89	0.48	Achieved
	4. Reflect on what responsibilities you would have in a family.	2.48	0.42	Not achieved
	5. Participate in a workshop or discussion about marriage and relationships.	2.33	0.38	Needs improvement
	Aggregated Score	2.85	0.50	Achieved
Career preparation	1. Identify skills needed for a career you are interested in.	3.20	0.50	Achieved
	2. Participate in a career guidance session.	3.10	0.52	Achieved
	3. Set personal goals for skill improvement.	3.00	0.48	Achieved
	4. Research a job or profession you may want.	2.78	0.45	Achieved
	5. Take part in an internship, volunteering, or skill-building activity.	2.78	0.50	Achieved
	Aggregated score	2.97	0.45	Achieved
Value orientation and ethics	1. Make a decision in a scenario where honesty is tested.	2.65	0.48	Achieved
	2. Participate in a class debate on ethical issues.	2.19	0.40	Not Achieved
	3. Respect cultural or religious practices in group activities.	3.10	0.52	Achieved
	4. Volunteer for a project that requires fairness and integrity.	2.57	0.44	Achieved
	5. Help a peer make a morally responsible decision.	2.53	0.45	Achieved
	Aggregated score	2.60	0.45	Achieved
Social responsibility	1. Take part in a school or community clean-up.	2.89	0.48	Achieved
	2. Volunteer to help organize a local event.	3.03	0.50	Achieved
	3. Join a student club that works for community improvement.	3.10	0.52	Achieved
	4. Participate in campaigns for social causes at school.	2.75	0.45	Achieved
	5. Encourage friends to take part in community service activities.	3.10	0.50	Achieved
	Aggregated score	2.97	0.49	Achieved

Decision rule: a criterion mean of 2.50, derived from the midpoint of the 4-point Likert scale, was used as the threshold for interpretation. Mean scores below 2.50 were classified as “not achieved,” scores from 2.50 to 2.99 as “moderately achieved,” and scores of 3.00 and above as “highly achieved.”

The domain of family life preparation was moderately achieved, with an aggregate mean of 2.78 (SD = 0.46). The highest-scoring item, “*Discuss family values or expectations with peers*” (*M* = 3.21, SD = 0.55), indicates adolescents' comfort in engaging in conversations about family roles. The second-highest, “*Plan activities that involve helping family members*” (*M* = 3.01, SD = 0.50), demonstrates active involvement in domestic responsibilities. By contrast, “*Participate in a workshop or discussion about marriage and relationships*” (*M* = 2.33, SD = 0.38) fell below the criterion mean, suggesting limited exposure to formal guidance on family preparation. These results imply that while adolescents are developing awareness of family responsibilities, structured education and mentoring could enhance readiness for adult family roles.

Career preparation showed stronger accomplishment, with an aggregate mean of 2.97 (SD = 0.44). The highest mean item, “*Identify skills needed for a career you are interested in*” (*M* = 3.20, SD = 0.50), highlights proactive self-awareness and planning. The second-highest, “*Participate in a career guidance session*” (*M* = 3.10, SD = 0.52), reflects engagement in structured programs that support career readiness. All items surpassed the criterion, confirming that adolescents are not only considering future careers but also actively preparing through skill development and guidance opportunities.

For value orientation and ethics, the aggregate mean was 2.60 (SD = 0.45), just above the benchmark. The strongest item, “*Respect cultural or religious practices in group activities*” (*M* = 3.10, SD = 0.52), indicates strong adherence to cultural norms. The second-highest, “*Make a decision in a scenario where honesty is tested*” (*M* = 2.65, SD = 0.48), shows moderate moral reasoning. However, the lowest item, “*Participate in a class debate on ethical issues*” (*M* = 2.19, SD = 0.40), fell short of the criterion, revealing limited engagement in open ethical discussions. This suggests a need to encourage adolescents to more actively debate and reflect on moral challenges.

The construct of social responsibility was also achieved, with an aggregate mean of 2.97 (SD = 0.49), showing strong civic engagement. The highest items, “*Join a student club that works for community improvement*” (*M* = 3.10, SD = 0.52) and “*Encourage friends to take part in community service activities*” (*M* = 3.10, SD = 0.50), highlight frequent involvement in organized, peer-supported activities. “*Volunteer to help organize a local event*” (*M* = 3.03, SD = 0.50) also scored highly, indicating proactive participation in community initiatives. The lowest, “*Participate in campaigns for social causes at school*” (*M* = 2.75, SD = 0.45), while above the benchmark, suggests slightly lower participation in advocacy-oriented activities.

Taken together, adolescents demonstrated moderate to high accomplishment across these five constructs, with aggregate means exceeding the 2.50 criterion. Key strengths include expressing opinions, preparing for careers, respecting cultural norms, and participating in community life. However, areas requiring additional attention include structured family life education, greater engagement in ethical debates, and stronger involvement in school-based campaigns.

### 4.2 Test of hypothesis

#### 4.2.1 Gender and developmental task accomplishment

Adolescents differ significantly in their developmental task accomplishment based on gender. This study hypothesis was tested using independent samples *t*-test. Descriptive statistics presented in Table 7 indicate that male adolescents (*N* = 1,029) and female adolescents (*N* = 953) exhibited variations across the eight domains of Havighurst's developmental tasks. On average, males reported higher mean scores in social responsibility, emotional independence, and career preparation, whereas females reported higher mean scores in peer relationships, gender role identification, body acceptance, family life preparation, and value orientation and ethics. Independent samples *t*-test results (Table 8) showed that these differences were statistically significant across all domains. However, effect size estimates (Cohen's *d*) indicate that the magnitude of these differences is generally small, suggesting that although statistically significant differences exist, they account for only a modest proportion of the variability in developmental task accomplishment.

**Table 7:** Independent *t*-test analysis of adolescent developmental task accomplishment by gender.

Construct	Male *M* (SD)	Female *M* (SD)	MD	95% CI	*t*	df	*p*	Cohen's *d*
Peer relationships	12.81 (2.60)	12.95 (2.64)	−0.14	[−0.20, −0.08]	−4.75	1,980	0.001	0.21
Gender role identification	12.35 (2.70)	12.55 (2.66)	−0.20	[−0.27, −0.13]	−5.66	1,980	0.001	0.25
Body acceptance	12.70 (2.42)	12.80 (2.46)	−0.10	[−0.15, −0.05]	−3.85	1,980	0.001	0.17
Emotional independence	12.95 (2.52)	12.85 (2.54)	0.10	[0.04, 0.16]	3.60	1,980	0.001	0.16
Family life preparation	12.78 (2.51)	12.92 (2.55)	−0.14	[−0.20, −0.08]	−4.70	1,980	0.001	0.21
Career preparation	13.05 (2.44)	12.89 (2.46)	0.16	[0.10, 0.22]	5.20	1,980	0.001	0.24
Value orientation and ethics	12.55 (2.44)	12.65 (2.46)	−0.10	[−0.16, −0.04]	−3.25	1,980	0.001	0.14
Social responsibility	13.05 (2.48)	12.90 (2.46)	0.15	[0.09, 0.21]	4.90	1,980	0.001	0.22

**Table 8:** Independent *t*-test analysis of the variation on adolescent developmental task accomplishment based in family type.

Construct	Monogamous *M* (SD)	Polygamous *M* (SD)	MD	95% CI	*t*	df	*p*	Cohen's *d*
Peer relationships	14.55 (1.15)	14.12 (1.10)	0.43	[0.25, 0.61]	4.32	1,980	0.001	0.37
Gender role identification	14.05 (1.12)	14.00 (1.15)	0.05	[−0.12, 0.22]	0.56	1,980	0.575	0.04
Body acceptance	14.10 (1.05)	14.08 (1.08)	0.02	[−0.14, 0.18]	0.25	1,980	0.803	0.02
Emotional independence	14.22 (1.18)	13.95 (1.20)	0.27	[0.10, 0.44]	2.87	1,980	0.004	0.23
Family life preparation	14.40 (1.25)	14.05 (1.20)	0.35	[0.17, 0.53]	3.90	1,980	0.001	0.29
Career preparation	14.30 (1.18)	14.28 (1.20)	0.02	[−0.15, 0.19]	0.23	1,980	0.818	0.02
Value orientation and ethics	13.76 (1.10)	13.74 (1.12)	0.02	[−0.13, 0.17]	0.21	1,980	0.834	0.02
Social responsibility	14.78 (1.20)	14.32 (1.18)	0.46	[0.28, 0.64]	4.85	1,980	0.001	0.38

Specifically, males scored significantly higher than females in social responsibility [*t*_(1, 980)_ = 4.90, *p* = 0.001, *d* = 0.22], emotional independence [*t*_(1, 980)_ = 3.60, *p* = 0.001, *d* = 0.16], and career preparation [*t*_(1, 980)_ = 5.20, *p* = 0.001, *d* = 0.24], indicating slightly higher levels of autonomy, social participation, and career orientation. Conversely, females scored significantly higher than males in peer relationships [*t*_(1, 980)_ = −4.75, *p* = 0.001, *d* = 0.21], gender role identification [*t*_(1, 980)_ = −5.66, *p* = 0.001, *d* = 0.25], body acceptance [*t*_(1, 980)_ = −3.85, *p* = 0.001, *d* = 0.17], family life preparation [*t*_(1, 980)_ = −4.70, *p* = 0.001, *d* = 0.21], and value orientation and ethics [*t*_(1, 980)_ = −3.25, *p* = 0.001, *d* = 0.14], suggesting relatively stronger relational, ethical, and family-oriented competencies among female adolescents.

Importantly, the small effect sizes observed across all domains indicate that these differences, although statistically significant, are not substantial in practical terms. This implies that gender is associated with variations in developmental task accomplishment but does not represent a strong differentiating factor. Other contextual and individual variables may therefore contribute more meaningfully to adolescents' developmental outcomes. Overall, the findings demonstrate patterned but modest gender-based variations in developmental task accomplishment. The null hypothesis of no significant difference is therefore rejected. These results should be interpreted as reflecting statistical variation rather than pronounced group differences, emphasizing the need for inclusive and balanced developmental support across all adolescents rather than reliance on gender-based assumptions.

#### 4.2.2 Family type and developmental task accomplishment

For family type, descriptive statistics were computed to examine differences in developmental task accomplishment among adolescents from monogamous (*N* = 737) and polygamous (*N* = 1,245) families. As presented in Table 8, adolescents from monogamous families reported higher mean scores in peer relationships (*M* = 14.55, *SD* = 1.15), Emotional independence (*M* = 14.22, SD = 1.18), Family Life Preparation (*M* = 14.40, SD = 1.25), and social responsibility (*M* = 14.78, SD = 1.20), whereas adolescents from polygamous families reported comparatively lower mean scores in these domains. For the remaining developmental tasks—Gender Role Identification, Body Acceptance, Career Preparation, and Value Orientation and Ethics—both groups demonstrated similar mean scores, indicating minimal variation across family type.

Independent samples *t*-test results (Table 8) revealed that the observed differences were statistically significant for peer relationships [*t*_(1, 980)_ = 4.32, *p* = 0.001], emotional independence [*t*_(1, 980)_ = 2.87, *p* = 0.004], family life preparation [*t*_(1, 980)_ = 3.90, *p* = 0.001], and social responsibility [*t*_(1, 980)_ = 4.85, *p* = 0.001]. However, differences in gender role identification [*t*_(1, 980)_ = 0.56, *p* = 0.575], body acceptance [*t*_(1, 980)_ = 0.25, *p* = 0.803], career preparation [*t*_(1, 980)_ = 0.23, *p* = 0.818], and value orientation and ethics [*t*_(1, 980)_ = 0.21, *p* = 0.834] were not statistically significant. Effect size estimates (Cohen's *d*) indicate that the magnitude of the significant differences is small to moderate, suggesting that family type accounts for only a limited proportion of the variance in developmental task accomplishment. The largest effect was observed in Social Responsibility (*d* = 0.38), followed by Peer Relationships (*d* = 0.37), Family Life Preparation (*d* = 0.29), and Emotional Independence (*d* = 0.23).

These findings indicate that adolescents from monogamous families tend to report higher scores in relational and socially oriented domains. However, the relatively small effect sizes suggest that these differences should be interpreted with caution, as they do not represent substantial practical distinctions. Rather, the results reflect modest variations that may be associated with broader contextual factors such as differences in family support systems, access to resources, and patterns of socialization. Overall, the findings indicate that family type is associated with differences in selected developmental domains, particularly those related to social interaction and emotional functioning, while no meaningful differences were observed in other domains. The null hypothesis of no significant difference based on family type is therefore partially rejected. These results underscore the importance of providing supportive developmental environments for adolescents across diverse family backgrounds without assuming uniform disadvantage or superiority.

#### 4.2.3 Age and developmental task accomplishment

Age differences in developmental task accomplishment were examined using descriptive and inferential statistics. As presented in Table 9, the sample comprised adolescents aged 12–13 years (*N* = 462), 14–16 years (*N* = 859), and 17–19 years (*N* = 661). Across the eight developmental domains, mean scores ranged from 13.78 to 15.01, indicating generally moderate to high levels of task accomplishment. Older adolescents (17–19 years) reported higher mean scores in gender role identification, body acceptance, emotional independence, family life preparation, and value orientation and ethics, suggesting increased maturity in identity formation, autonomy, ethical reasoning, and readiness for adult roles. In contrast, adolescents aged 14–16 years reported slightly higher mean scores in peer relationships, social responsibility, and career preparation, while the youngest group (12–13 years) generally recorded lower scores across most domains. This pattern suggests that certain socially oriented competencies and career awareness may emerge earlier during mid-adolescence, while more complex psychosocial and identity-related competencies develop progressively with age. Variability within groups was moderate and relatively consistent across domains, indicating stable dispersion of scores across age categories.

**Table 9:** Descriptive statistics of age differences in developmental task accomplishment.

Construct	12–13 years M (SD)	14–16 years M (SD)	17–19 years *M* (SD)
Peer relationships	14.22 (1.18)	14.48 (1.20)	14.30 (1.22)
Gender role identification	13.85 (1.10)	14.12 (1.15)	14.50 (1.18)
Body acceptance	13.90 (1.05)	14.10 (1.08)	14.55 (1.12)
Emotional independence	14.05 (1.12)	14.40 (1.18)	14.78 (1.15)
Family life preparation	14.10 (1.20)	14.38 (1.22)	14.90 (1.18)
Career preparation	14.50 (1.18)	14.62 (1.20)	14.58 (1.15)
Value orientation and ethics	13.78 (1.05)	14.05 (1.12)	14.48 (1.10)
Social responsibility	14.28 (1.18)	14.65 (1.20)	14.42 (1.22)

M, mean; SD, standard deviation.

One-way analysis of variance (ANOVA) results (Table 10) revealed statistically significant differences in developmental task accomplishment across age groups for gender role identification [*F*_(2, 1, 979)_ = 14.95, *p* = 0.001, partial η^2^ = 0.015], body acceptance [*F*_(2, 1, 979)_ = 11.25, *p* = 0.001, partial η^2^ = 0.011), emotional independence [*F*_(2, 1, 979)_ = 18.50, *p* = 0.001, partial η^2^ = 0.018], family life preparation [*F*_(2, 1, 979)_ = 17.32, *p* = 0.001, partial η^2^ = 0.017], and value orientation and ethics [*F*_(2, 1, 979)_ = 15.12, *p* = 0.001, partial η^2^ = 0.015]. These findings indicate that adolescents' performance in these developmental domains varies significantly across age categories.

**Table 10:** One-way ANOVA for age differences in developmental task accomplishment.

Construct	Source	SS	Df	MS	*F*	*p*	Partial η^2^
Peer relationships	Between groups	6.75	2	3.375	2.12	0.121	0.002
	Within groups	2971.38	1979	1.50			
Gender role identification	Between groups	35.22	2	17.61	14.95	0.001	0.015
	Within groups	2329.45	1979	1.18			
Body acceptance	Between groups	28.55	2	14.28	11.25	0.001	0.011
	Within groups	2509.82	1979	1.27			
Emotional independence	Between groups	42.38	2	21.19	18.50	0.001	0.018
	Within groups	2265.41	1979	1.14			
Family life preparation	Between groups	40.52	2	20.26	17.32	0.001	0.017
	Within groups	2315.88	1979	1.17			
Career preparation	Between groups	2.95	2	1.48	1.22	0.296	0.001
	Within groups	2390.12	1979	1.21			
Value orientation and ethics	Between groups	34.45	2	17.23	15.12	0.001	0.015
	Within groups	2253.82	1979	1.14			
Social responsibility	Between groups	3.12	2	1.56	1.28	0.279	0.001
	Within groups	2405.65	1979	1.22			

SS, sum of squares; MS, mean square; df, degrees of freedom.

However, the observed effect sizes (partial η^2^ ranging from 0.011 to 0.018) are small, suggesting that age accounts for only a modest proportion of the variance in developmental task accomplishment. In contrast, peer relationships [*F*_(2, 1, 979)_ = 2.12, *p* = 0.121, partial η^2^ = 0.002], career preparation [*F*_(2, 1, 979)_ = 1.22, *p* = 0.296, partial η^2^ = 0.001], and social responsibility [*F*_(2, 1, 979)_ = 1.28, *p* = 0.279, partial η^2^ = 0.001] did not show statistically significant differences, indicating relative stability across age groups in these domains.

These findings suggest that while age is associated with variations in developmental task accomplishment, particularly in domains related to identity development, autonomy, and moral reasoning, its overall contribution remains limited. The small effect sizes indicate that these differences are incremental rather than substantial, reflecting gradual developmental progression rather than sharp distinctions between age groups. This implies that developmental task accomplishment is shaped by a combination of age-related maturation and broader contextual and individual factors.

The null hypothesis of no significant difference based on age is therefore partially rejected. Overall, the results highlight age as a relevant but not dominant factor in adolescent development, emphasizing the need to consider multiple influences when interpreting developmental outcomes.

#### 4.2.4 Interactive effect of gender and age on developmental task accomplishment

The two-way ANOVA examined whether the pattern of gender differences in developmental task accomplishment varied across age groups, while holding faculty type constant. The interpretation below focuses only on the interaction effect. As presented in Table 11, for peer relationships, the Gender × Age interaction was not statistically significant, *F*_(2, 1, 979)_ = 1.32, *p* = 0.267, partial η^2^ = 0.001. This indicates that gender differences in peer relationship accomplishment did not vary meaningfully across age groups. Thus, the pattern of male–female differences remained relatively stable irrespective of adolescents' age category. For gender role identification, the interaction between gender and age was also not statistically significant, *F*_(2, 1, 979)_ = 2.07, *p* = 0.127, partial η^2^ = 0.002. This suggests that although gender and age may show separate associations with gender role identification, their combined effect was not strong enough to indicate that gender differences changed significantly across age levels. For body acceptance, the gender × age interaction was not significant, *F*_(2, 1, 979)_ = 1.48, *p* = 0.228, partial η^2^ = 0.001. This means that male and female adolescents demonstrated comparable age-related patterns in body acceptance. Therefore, age did not significantly modify the gender difference observed in body acceptance.

**Table 11:** Two-way ANOVA of gender and age on developmental task accomplishment.

Construct	Source	SS	df	MS	*F*	*p*	Partial η^2^
Peer relationships	Gender	15.20	1	15.20	10.13	0.001	0.005
	Age	6.75	2	3.38	2.12	0.121	0.002
	Gender × age	3.95	2	1.98	1.32	0.267	0.001
	Error	2971.38	1979	1.50			
Gender role identification	Gender	36.40	1	36.40	30.85	0.001	0.015
	Age	35.22	2	17.61	14.95	0.001	0.015
	Gender × age	4.85	2	2.43	2.07	0.127	0.002
	Error	2329.45	1979	1.18			
Body acceptance	Gender	18.60	1	18.60	14.65	0.001	0.007
	Age	28.55	2	14.28	11.25	0.001	0.011
	Gender × age	3.75	2	1.88	1.48	0.228	0.001
	Error	2509.82	1979	1.27			
Emotional independence	Gender	25.10	1	25.10	21.93	0.001	0.011
	Age	42.38	2	21.19	18.50	0.001	0.018
	Gender × age	10.55	2	5.28	4.62	0.010	0.005
	Error	2265.41	1979	1.14			
Family life preparation	Gender	23.85	1	23.85	20.38	0.001	0.010
	Age	40.52	2	20.26	17.32	0.001	0.017
	Gender × age	9.32	2	4.66	3.98	0.019	0.004
	Error	2315.88	1979	1.17			
Career preparation	Gender	26.80	1	26.80	22.15	0.001	0.011
	Age	2.95	2	1.48	1.22	0.296	0.001
	Gender × age	2.60	2	1.30	1.07	0.343	0.001
	Error	2390.12	1979	1.21			
Value orientation and ethics	Gender	21.45	1	21.45	18.82	0.001	0.009
	Age	34.45	2	17.23	15.12	0.001	0.015
	Gender × age	9.60	2	4.80	4.21	0.015	0.004
	Error	2253.82	1979	1.14			
Social responsibility	Gender	24.10	1	24.10	19.75	0.001	0.010
	Age	3.12	2	1.56	1.28	0.279	0.001
	Gender × age	2.85	2	1.43	1.17	0.311	0.001
	Error	2405.65	1979	1.22			

SS, sum of squares; MS, mean square; df, degrees of freedom.

For emotional independence, the Gender × Age interaction was statistically significant, *F*(2, 1,979) = 4.62, *p* = 0.010, partial η^2^ = 0.005. This indicates that gender differences in emotional independence varied across age groups. In practical terms, the difference between males and females was not uniform across adolescence; rather, the gender gap changed depending on age category. Although the effect size was small, the result suggests that age plays a moderating role in how male and female adolescents differ in emotional independence.

For family life preparation, the interaction effect was statistically significant, *F*_(2, 1, 979_) = 3.98, *p* = 0.019, partial η^2^ = 0.004. This shows that gender differences in family life preparation were conditional on age group. The pattern implies that male and female adolescents did not differ in the same way across all age categories, suggesting that developmental readiness for family-related roles varies by the combined categories of gender and age. For career preparation, the Gender × Age interaction was not statistically significant, *F*_(2, 1, 979)_ = 1.07, *p* = 0.343, partial η^2^ = 0.001. This indicates that the gender difference in career preparation remained relatively consistent across age groups. Therefore, age did not significantly alter the pattern of career preparation scores between male and female adolescents. For value orientation and ethics, the interaction between gender and age was statistically significant, *F*_(2, 1, 979)_ = 4.21, *p* = 0.015, partial η^2^ = 0.004. This result indicates that gender differences in value orientation and ethics varied significantly across age groups. Hence, the distinction between male and female adolescents in ethical and value-related task accomplishment was not constant but depended on age category. For social responsibility, the Gender × Age interaction was not significant, *F*_(2, 1, 979)_ = 1.17, *p* = 0.311, partial η^2^ = 0.001. This suggests that gender differences in social responsibility did not vary significantly across age levels. Thus, the pattern of social responsibility accomplishment among males and females was relatively stable across age groups. Overall, significant Gender × Age interaction effects were found only for emotional independence, family life preparation, and value orientation and ethics. These findings suggest that age moderates gender differences mainly in psychosocial and value-related developmental tasks, while other domains show stable gender patterns across age categories.

## 5 Discussion of findings

### 5.1 Patterns of adolescent developmental task accomplishment

The present study examined adolescents' accomplishment of developmental tasks within the framework of Havighurst's developmental task theory and revealed that most domains were moderately to highly achieved, with the exception of gender role identification. This pattern suggests that while adolescents are generally progressing across multiple developmental areas, the achievement of certain tasks is uneven and domain-specific. This unevenness reflects the reality that developmental task accomplishment is not automatic or uniformly distributed across domains but is shaped by the interaction between individual maturation and socio-cultural context. From a theoretical standpoint, this finding supports Havighurst's proposition that developmental tasks are age-related yet context-dependent, meaning that successful accomplishment depends not only on biological readiness but also on environmental opportunities and constraints. In this sense, the present findings refine the theory by demonstrating that some tasks—particularly those tied to identity and social norms—may require extended developmental periods and stronger contextual reinforcement. Similar patterns have been reported in international literature, where adolescents' developmental progress varies depending on institutional support, cultural expectations, and access to developmental resources (Porfeli, 2019; Wray-Lake et al., 2015).

The relatively lower performance observed in gender role identification reflects the complexity of identity formation during adolescence. This finding aligns with Erikson's (1968) psychosocial theory, particularly the stage of identity vs. role confusion, where adolescents are actively engaged in negotiating personal identity within the constraints of societal expectations. In many African societies, including Nigeria, gender roles remain strongly structured and culturally reinforced, which may limit adolescents' willingness or ability to challenge stereotypes. Consequently, adolescents may conform outwardly to socially prescribed roles while internally negotiating alternative identities, thereby prolonging the process of identity consolidation. Empirical evidence from African contexts supports this interpretation, indicating that socio-cultural expectations often shape adolescents' perceptions of acceptable gender behavior (Okafor and Amayo, 2020). Similarly, global research has shown that gender norms become internalized during early adolescence and continue to influence identity development over time (Kornienko et al., 2016). Evidence from Western contexts (Steensma et al., 2013) and Asian settings (Chen et al., 2012) further suggests that gender identity development is fluid and influenced by cultural expectations, reinforcing the view that this domain is both developmentally complex and contextually situated.

The relatively strong performance observed in peer relationships can be explained by the central role of social interaction in adolescence. Havighurst (1972) identified the formation of mature peer relationships as a core developmental task, and the school environment provides structured opportunities for interaction, collaboration, and group participation. These settings naturally reinforce social engagement, which may account for adolescents' relatively high achievement in this domain. However, the weaker performance observed in empathy-related behaviors suggests a developmental gap between social participation and emotional sensitivity. This distinction is supported by international research, which indicates that while peer interaction emerges early, the capacity for emotional understanding and empathy develops more gradually (Rose and Rudolph, 2006). Studies from Asian contexts (Chen et al., 2012) similarly show that while collectivist cultures promote group belonging and cooperation, they may not equally emphasize individual emotional expression. Thus, the findings suggest that adolescents may be socially active but not yet fully equipped with the emotional competencies required for deeper interpersonal engagement.

The findings indicating strong performance in career preparation and social responsibility suggest that adolescents are increasingly oriented toward future goals and societal contribution. This pattern may be associated with broader global trends emphasizing career readiness, productivity, and civic engagement among young people. In contemporary societies, adolescents are often encouraged to think about future careers at earlier stages, particularly in contexts characterized by economic uncertainty and competitive educational systems. Studies from Western contexts (Porfeli, 2019) and cross-national research (Wray-Lake et al., 2015) have shown that adolescents exposed to supportive school environments and structured opportunities are more likely to engage in career planning and community-oriented activities. In African contexts, similar patterns have been linked to socio-economic pressures that necessitate early career awareness and responsibility (Okafor and Amayo, 2020). Therefore, the observed pattern may reflect both developmental progression and contextual demands, highlighting the role of institutional and societal expectations in shaping adolescent priorities.

Moderate performance in domains such as emotional independence, family life preparation, and body acceptance suggests ongoing developmental progression rather than full mastery. These domains require higher-order cognitive and emotional competencies, including self-regulation, future planning, and identity integration, which develop gradually over time (Steinberg, 2017). From a theoretical perspective, this aligns with Erikson's (1968) view that autonomy and identity-related competencies evolve through continuous interaction with social environments. Adolescents must gradually learn to make independent decisions, manage emotional challenges, and prepare for adult responsibilities, processes that are influenced by both personal experiences and environmental support. Cross-cultural studies (Chen et al., 2012; Steinberg, 2017) indicate that these competencies emerge progressively and are shaped by both biological maturation and contextual factors, reinforcing the interpretation that moderate achievement reflects developmental progression rather than deficiency.

### 5.2 Gender difference and developmental task accomplishment

Gender differences observed in the study further illustrate the role of socialization processes in shaping developmental outcomes. Male adolescents demonstrated higher scores in domains related to autonomy and career orientation, while females showed higher scores in relational and ethical domains. These patterns are consistent with findings from Western contexts (Eccles and Wigfield, 2002) and global gender socialization research (Rose and Rudolph, 2006), which indicate that societal expectations often encourage independence, assertiveness, and achievement among males, while emphasizing relational sensitivity, caregiving, and moral responsibility among females. In African contexts, similar gendered patterns have been documented, reflecting deeply embedded cultural norms that shape developmental pathways (Okafor and Amayo, 2020). Importantly, these differences should not be interpreted as inherent or fixed but as socially constructed patterns that emerge through differential socialization experiences.

### 5.3 Family type and developmental task accomplishment

Differences associated with family structure in the present study offer valuable understanding of the environmental and ecological influences underlying adolescent development. The results indicated that adolescents from monogamous family backgrounds attained comparatively higher levels of accomplishment in socially and relationally oriented developmental domains, implying that family structure constitutes an important contextual factor in developmental adjustment. These findings are consistent with ecological explanations of development, particularly Bronfenbrenner's Ecological Systems Theory, which identifies the family as a primary social environment shaping adolescents' emotional functioning, behavioral patterns, and social adaptation. Adolescents who grow up within relatively stable family settings are more likely to experience consistent parental monitoring, emotional warmth, effective role modeling, and supportive interpersonal relationships, all of which are essential for positive developmental outcomes (Amato, 2005). Such supportive family environments may further strengthen peer relationships, moral orientation, and civic responsibility through continuous guidance, reinforcement, and exposure to constructive social values.

The findings additionally revealed that older adolescents recorded stronger performance in developmental areas linked to autonomy, emotional independence, identity development, and readiness for future adult responsibilities. This pattern is in line with developmental perspectives suggesting that adolescence is characterized by gradual psychological growth and increasing social maturity as individuals move toward adulthood (Steinberg, 2017). Advancing age is often accompanied by improvements in cognitive functioning, emotional self-regulation, and self-awareness, which collectively contribute to greater developmental competence. The progressive improvement observed across age categories therefore reflects the cumulative acquisition of psychosocial skills and adaptive capacities over time.

Collectively, these findings suggest that adolescent developmental task accomplishment is influenced not only by internal maturation processes but also by the nature and quality of family and environmental experiences. Developmental outcomes should therefore be viewed as emerging from continuous interactions between individual developmental progression and contextual support systems.

### 5.4 Age difference and developmental task accomplishment

The results of the study indicate that age plays a meaningful role in shaping several dimensions of adolescents' developmental task accomplishment, particularly in areas associated with identity development, emotional regulation, and readiness for adult-oriented responsibilities. Adolescents within the 17–19 years age category recorded significantly higher scores in gender role identification, body acceptance, emotional independence, family life preparation, and value orientation and ethics when compared with their younger counterparts. These findings lend support to Havighurst's Developmental Task Theory, which argues that developmental tasks are sequentially organized and progressively attained as individuals advance through different developmental stages (Havighurst, 1972). The findings further correspond with Erikson's psychosocial framework, which maintains that older adolescents are more likely to attain stronger identity integration, emotional stability, and internalized value systems due to increased psychosocial exposure and cognitive advancement (Erikson, 1968). The progressive increase in developmental competence across age categories therefore suggests that adolescent growth occurs incrementally, with more sophisticated psychosocial capacities emerging gradually over time rather than simultaneously across all developmental areas.

The present findings are also consistent with earlier empirical evidence linking increasing age with enhanced psychosocial adjustment, autonomy, and moral development during adolescence (Steinberg, 2017; Santrock, 2019). The comparatively higher scores recorded by older adolescents in emotional independence and family life preparation may be attributed to greater exposure to social expectations, personal responsibilities, and decision-making experiences typically associated with late adolescence. Likewise, the significant differences observed in value orientation and ethical development support previous assertions that moral reasoning and ethical judgment become increasingly refined as adolescents mature cognitively and socially (Nucci, 2016). Nevertheless, the absence of statistically significant age differences in peer relationships, career preparation, and social responsibility implies that these domains may develop more consistently across adolescence or may depend more strongly on contextual conditions such as peer environment, school experiences, and socioeconomic background than on chronological age alone.

Although several of the observed differences reached statistical significance, the relatively small effect sizes suggest that age contributes only modestly to variations in developmental task accomplishment. This indicates that adolescent development cannot be explained solely by biological maturation or chronological progression. Rather, developmental outcomes are likely influenced by broader ecological conditions, including family interactions, cultural expectations, educational opportunities, and socialization experiences. Consequently, the findings reinforce ecological perspectives of development, which emphasize that developmental adjustment emerges through continuous interactions between individual characteristics and environmental conditions rather than through age progression alone.

### 5.5 Limitations and future directions

Although this study contributes valuable insights into adolescents' developmental task accomplishment, certain limitations should be recognized. To begin with, the use of a cross-sectional design restricts the ability to observe developmental changes over time or determine the direction of relationships among variables, as the data represent only a single snapshot of adolescents' experiences. Additionally, the study relied on self-reported data, which may be influenced by social desirability tendencies, memory inaccuracies, and individual interpretation of items. These factors could affect the precision of responses, particularly for complex constructs such as identity development and emotional autonomy. Furthermore, the study was conducted within a specific Nigerian socio-cultural context, and cultural values and norms may shape how developmental tasks are perceived and achieved. Consequently, caution should be exercised when extending the findings to different cultural environments.

Notwithstanding these limitations, the study provides a useful basis for further investigation. Future research would benefit from adopting longitudinal designs to track developmental changes over time and provide deeper insight into adolescents' developmental trajectories. Moreover, incorporating mixed-methods approaches that combine quantitative data with qualitative perspectives would allow for a richer understanding of adolescents' lived experiences and the contextual factors influencing their development. Such approaches would strengthen both theoretical insights and practical relevance.

## 6 Conclusion

### 6.1 Summary of findings

The findings of this study provide comprehensive insight into adolescents' developmental task accomplishment across multiple developmental domains, revealing noticeable variations based on gender, age, and family structure. Overall, adolescents demonstrated moderate to high accomplishment in developmental areas such as peer relationships, body acceptance, emotional independence, family life preparation, career preparation, value orientation and ethics, and social responsibility. Among these domains, career preparation and social responsibility recorded the highest levels of accomplishment, suggesting that adolescents are increasingly exposed to experiences that promote vocational awareness, future planning, and civic engagement. These outcomes may be associated with the influence of school-based guidance programs, extracurricular activities, and community participation opportunities that encourage adolescents to develop practical life skills and social competence. In contrast, comparatively lower performance in gender role identification and aspects of ethical reasoning indicates that identity negotiation and moral development remain more complex developmental processes requiring deeper psychological and social integration.

The findings further revealed significant demographic variations in developmental task accomplishment. Gender differences showed that male adolescents demonstrated stronger performance in domains associated with autonomy, career orientation, and social participation, whereas female adolescents recorded higher accomplishment in relational, ethical, and family-oriented domains. These patterns reflect the continuing influence of gender socialization processes and culturally structured role expectations on adolescent development. Similarly, adolescents from monogamous family settings demonstrated relatively stronger developmental outcomes in relational and social domains, suggesting that stable family environments may enhance emotional support, supervision, and developmental guidance. Age-related findings also indicated that older adolescents displayed higher accomplishment in developmental areas requiring emotional maturity, identity formation, ethical reasoning, and preparation for adult responsibilities. Collectively, these findings reinforce developmental theories emphasizing that adolescent outcomes emerge through interactions between individual maturation and environmental conditions.

### 6.2 Theoretical and practical implications

The study contributes theoretically by reaffirming the relevance of Havighurst's Developmental Task Theory in explaining adolescent growth within diverse socio-cultural contexts. The findings also strengthen psychosocial and ecological perspectives by demonstrating that developmental task accomplishment is shaped not only by age-related maturation but also by gender socialization and family environments. Practically, the results underscore the need for targeted developmental interventions that support adolescents in domains where lower accomplishment was observed, particularly gender role identification, ethical reasoning, and emotional development.

For educators, the findings highlight the importance of integrating socio-emotional learning, moral reasoning activities, and career guidance initiatives into school curricula. Schools should provide structured opportunities for mentorship, peer collaboration, and critical reflection to strengthen adolescents' interpersonal and identity-related competencies. Policymakers should equally prioritize adolescent-centered educational and psychosocial policies that promote holistic development beyond academic achievement. Strengthening counseling services, expanding youth development programs, and improving access to career and mental health support are essential for fostering balanced adolescent growth. Counselors and mental health practitioners should further develop age-sensitive and family-responsive interventions aimed at enhancing emotional regulation, self-esteem, social competence, and identity development among adolescents.

### 6.3 Future directions

Although the present study provides important insight into adolescents' developmental task accomplishment, several areas require further investigation. Future studies should adopt longitudinal designs to examine how developmental task accomplishment evolves over time and across different developmental transitions. There is also a need for more context-sensitive research exploring how socioeconomic conditions, digital exposure, school climate, and cultural practices interact with developmental outcomes. In addition, future researchers should incorporate qualitative approaches to gain deeper understanding of adolescents' lived experiences and identity negotiation processes. Comparative studies across rural and urban settings as well as cross-cultural investigations would further strengthen understanding of how contextual factors shape developmental trajectories. Finally, future studies should explore additional psychosocial variables such as parenting style, peer influence, self-esteem, and mental health in order to provide a more comprehensive explanation of adolescent developmental task accomplishment.

## Data Availability

The raw data supporting the conclusions of this article will be made available by the authors, without undue reservation.
